# Decoding clinical diversity in monogenic TGFBR1 and TGFBR2 mutations: insights into the interplay of molecular mechanisms and hypomorphicity

**DOI:** 10.3389/fcell.2025.1580274

**Published:** 2025-06-19

**Authors:** Fadia Abu-Sailik, Nesrin Gariballa, Bassam R. Ali

**Affiliations:** ^1^ Department of Genetics and Genomics, College of Medicine and Health Sciences, United Arab Emirates University, Al-Ain, United Arab Emirates; ^2^ ASPIRE Precision Medicine Research Institute, Abu Dhabi, United Arab Emirates University, Al-Ain, United Arab Emirates

**Keywords:** TGFBR1, TGFBR2, LDS, MFS2, TAAD, MSSE, dominant-negative, ERAD

## Abstract

Several autosomal-dominant monogenic disorders have been conclusively associated with mutations in TGFBR1 and TGFBR2, key receptors of the Transforming Growth Factor-β (TGFβ) signaling pathway. Although these disorders share a common cardiovascular connective tissue manifestation, different mutations present with strikingly distinctive clinical presentations leading to distinct disorders, including Loeys-Dietz syndrome Marfan syndrome type 2 (MFS2), and Thoracic Aortic Aneurysms and Dissections (TAAD). In addition, some mutations lead to Shprintzen-Goldberg syndrome which is characterized by skeletal deformities and intellectual disabilities in addition to the cardiovascular involvement, or vascular Ehlers-Danlos Syndrome (vEDS) that is associated with spontaneous rupture of the main arteries and internal organs. Furthermore, Multiple Self-healing Squamous Epithelioma (MSSE), a rare familial skin cancer, is linked to mutations in these genes. This significant phenotypic variability observed in these disorders could be attributed to various factors, ranging from the nature of the mutation including its location within the protein, the variable functional impact of the mutations (hypomorphicity), the level of disruption to the intricate interactions between signaling pathways, and the influence of modifier genes or environmental factors. In addition to haploinsufficiency, the impairment of TGFβ signaling could be exacerbated in other scenarios, such as the dominant-negative effects, in which a mutant allele disrupts the normal activity of the wild-type protein by forming non-functional receptor oligomers, hindering their trafficking. This review sheds light on these hereditary disorders, highlighting the broad spectrum of their clinical presentations associated with mutations in the same gene, their pathophysiology, and underlying molecular mechanisms. Most crucially, it underscores the critical gaps in our current understanding while proposing compelling directions for future research. This review also emphasizes the pressing need to unravel the complex genotype-phenotype correlations, which could pave the way for more precise diagnostic and therapeutic strategies.

## 1 Introduction

Transforming Growth Factor-β Receptor 1 (TGFBR1) and Transforming Growth Factor-β Receptor 2 (TGFBR2) are key components of the TGFβ signaling pathway, a fundamental and well-conserved signaling pathway that regulates numerous biological processes. The two receptors are transmembrane proteins with a highly conserved Serine/Threonine kinase domain (STK) and form heterotetrameric complexes upon binding to TGFβ ligands (Massagué et al., 1994). TGFBR2 plays a vital role in activating TGFBR1 by phosphorylating specific serine and threonine residues. Once activated, TGFBR1 instigates the signaling cascades by phosphorylating downstream proteins that regulate gene expression and various cellular responses (Wrana et al., 1994).

Many genes encoding components of the TGFβ signaling pathway have been linked to a wide range of human monogenic disorders, each with distinctive clinical manifestations (Gariballa and Ali, 2020). For example, mutations in the *TGFBR1* or *TGFBR2* genes often exhibit a spectrum of syndromes affecting various organs and presenting with a wide range of symptoms, even when the mutations affect the same domain or the receptor (Loeys et al., 2006; Wharton and Derynck, 2009). Variation in phenotypes linked to mutations in a single gene arises from the contributions of various factors, including the type of mutation and its associated molecular mechanisms, the gene’s involvement in various signaling pathways, genetic background, including modifier genes, environmental influences, and epigenetic changes. This intricate interplay enables a single gene to be associated with multiple diseases, each presenting a distinct set of clinical characteristics. *TGFBR1* and *TGFBR2* are typical examples of this well-known yet under-investigated genetic pathogenesis phenomenon.

The phenotypic variability seen in disorders linked to TGFBR1 and TGFBR2 leads to a wide array of disease presentations, ranging from isolated vascular complications to comprehensive syndromic conditions like Loeys-Dietz syndrome (LDS), which is the most prominent of these disorders and characterized by aortic aneurysms, arterial tortuosity, skeletal deformities, craniofacial abnormalities, and skin manifestations (Loeys et al., 2005). LDS is subclassified into several types, depending on the specific gene mutation and the clinical presentation, with Types 1 through 5 being the most commonly recognized (Loeys et al., 2006; Schepers et al., 2018). Additionally, Marfan syndrome type 2 (MFS2), which arises from mutations in *TGFBR1* or *TGFBR2*, presents with features resembling the classical Marfan syndrome (MFS) caused by *FBN1* mutations, such as aortic dilation and skeletal abnormalities, although it lacks some of the broader systemic features typical of MFS (Mizuguchi et al., 2004). Thoracic Aortic Aneurysms and Dissections (TAAD) can also occur in a familial pattern due to *TGFBR1* or *TGFBR2* variations, leading to vascular complications independent of the systemic features seen in syndromic forms like LDS (Boileau et al., 2012). Moreover, Shprintzen-Goldberg syndrome (SGS), a disorder involving craniosynostosis, intellectual disability, and skeletal abnormalities, has been associated with mutations in TGFBR2, though it presents with distinct craniofacial and neurological features compared to LDS (Van Steensel et al., 2008). While Vascular Ehlers-Danlos Syndrome (vEDS) is primarily linked to mutations in *COL3A1*, there is some phenotypic overlap with LDS, particularly concerning vascular fragility, prompting research into potential roles of *TGFBR2* mutations in vEDS-like presentations (Weerakkody et al., 2016). Additionally, loss-of-function mutations in *TGFBR1* can cause a rare familial type of skin cancer known as Multiple Self-healing Squamous Epithelioma (MSSE) (Goudie, 2020). Collectively, these conditions highlight the critical role of TGFBR1 and TGFBR2 in maintaining the structural integrity of connective tissues, particularly in the cardiovascular system, and underscore the diverse pathogenic outcomes resulting from dysregulation of the TGFβ signaling pathway. However, the underlying mechanisms of this variability in the clinical presentations are under-investigated and, therefore, warrant further research.

Genetic diseases caused by variations in TGFβ signaling components are profoundly impacted by haploinsufficiency, hypomorphicity, and dominant-negative effects (Lindsay and Dietz, 2011; Gariballa et al., 2024a; Gariballa et al., 2024b). Haploinsufficiency arises when half the normal dose of a gene product is not enough for normal biological processes, often resulting in wide-ranging impacts contingent on the gene and cellular setting (Veitia, 2002). In addition, hypomorphic mutations result in a partial loss of gene function, allowing for some residual activity; the extent of this remaining function can influence the severity of the phenotype, creating a gradient or a spectrum of impact that is typically less severe than that seen with complete loss-of-function mutations (Meneely, 2020). Moreover, dominant-negative effects complicate the clinical picture further, as the mutated proteins can hinder the wild-type (WT) protein function by forming non-functional complexes that aggravate the loss of normal protein activity and disrupt critical cellular processes (Gerasimavicius et al., 2022).

This review will explore monogenic disorders linked to mutations in TGFBR1 and TGFBR2, as well as the various clinical presentations of these syndromes. We anticipate that the pathophysiology of disorders related to a significant number of TGFBR1 and TGFBR2 mutations is notably influenced by impaired receptor trafficking coupled with the hypomorphicity or, in some cases, dominant-negative effects. To identify and degrade misfolded proteins, the endoplasmic reticulum-associated degradation (ERAD) mechanism plays an essential role in protein quality control; however, it has also been implicated in disease pathogenesis (Zhao and Ackerman, 2006; Kaneko et al., 2017). The occurrence of several phenotypes resulting from individual mutations in the same gene may be explained by the interaction of hypomorphicity in terms of loss of function, dominant-negative interference, and ER quality control mechanisms (ERQCM). We anticipate that a better understanding of the molecular and cellular mechanisms underlying these disorders will lead to more personalized and efficient treatments that can enhance patient outcomes.

## 2 TGFβ signaling components

TGFβ signaling is well conserved and has a primary role in driving developmental programs and governing cellular behavior. This was demonstrated by the various effects of TGFβ-related cytokines on cellular homeostasis, regeneration, proliferation, and differentiation in context-dependent and cell-type-specific manner, as well as organ-specific morphogenesis (Massagué, 2012; Morikawa et al., 2016).

Thirty-three human genes belong to the TGFβ family, most of which encode secreted polypeptides with a dimeric structure stabilized by disulfide bonds. These genes are ubiquitously expressed in diverse tissues across both vertebrates and invertebrates (Derynck, 2008; Moustakas and Heldin, 2009). In addition to TGFβs, these include Bone Morphogenetic Proteins (BMPs), Growth and Differentiation factors (GDFs), Anti-Mullerian hormone (AMH), Activins, Nodal, and Inhibins (Wakefield and Hill, 2013).

Despite the abundance of TGFβ ligands, there are a total of twelve TGFβ receptors in humans and other mammals. Among these, five are classified as type II receptors, including TGFBR2, Activin A Receptor Type 2A (ACVR2A), Activin A Receptor Type 2B (ACVR2B), Bone morphogenetic protein receptor Type 2 (BMPR2), and Anti-Müllerian Hormone Receptor Type 2 (AMHR2). Likewise, there are seven type I receptors known as Activin Receptor-Like Kinases 1-7 (ALK1-7) (Moustakas and Heldin, 2009). These receptors are known for their cytoplasmic kinase domain, which exhibits robust serine/threonine kinase activity and relatively weaker tyrosine kinase activity. This unique combination of kinase activities classifies them as dual-specificity kinases (Heldin and Moustakas, 2016). In addition, the selectivity of ligand-receptor combinations is determined by interactions with adjacent or distant molecule surfaces. For example, TGFβ ligands specifically interact with both the type I receptor TGFBR1 (also referred to as ALK5 or TβRI) and the type II receptor TGFBR2 (also referred to as TβRII) (Shi and Massagué, 2003). In addition to type I and type II receptors, there are type III receptors (also known as co-receptors) present on the cell surface, including endoglin and the proteoglycan betaglycan (TGFBR3; TβRIII), that play roles in regulating TGFβ signaling in mammals (López-Casillas et al., 1993; Vander Ark et al., 2018). Unlike Type I and Type II receptors, the co-receptors lack a functional enzymatic motif and have reduced binding affinities for TGFβ family members, although they are more abundant compared to the signaling receptors (Nickel et al., 2018).

The transmission of intracellular signaling is facilitated by complexes of type I and II receptors and downstream intracellular effectors known as the mothers against decapentaplegic (Smad) proteins. Following binding, ligands assemble the two types of receptors: type I, which is involved in signal propagation, and type II, which phosphorylates and activates type I receptors on specific serine and threonine residues in the GS domain, therefore stabilizing their heterotetrameric structure. Activated type I receptors, in turn, propagate signals through the phosphorylation of carboxy-terminal serine residues of receptor-regulated (R-) Smads, leading to their dimerization (Feng and Derynck, 2005). Cell types typically exhibit phosphorylation of specific Smad proteins in response to different signaling molecules. TGFβs and activins induce phosphorylation of Smad2 and Smad3, which are known as activin/TGFβ-specific R-Smads. On the other hand, BMPs induce phosphorylation of Smad1, Smad5, and Smad8, which are referred to as BMP-specific R-Smads (Feng and Derynck, 2005; Massagué et al., 2005). Once activated, R-Smads combine with a common mediator, Co-Smad, Smad4, and create trimeric complexes of two R-Smads and a single Smad4, which are subsequently translocated to the nucleus and collaborate in tandem with other transcription factors, coactivators, and corepressors to regulate target gene expression in a cell-type-specific manner (Moustakas and Heldin, 2009).

To accomplish precise regulation of signaling specificity of TGFβ family members, it is necessary to implement negative regulation of the Smad signal, There is a distinct subclass of Smads called Inhibitory (I-Smads); Smad6 and 7, which play a crucial role in this context (Itoh and ten Dijke, 2007; Moustakas and Heldin, 2009). It was first discovered that I-Smads inhibit R-Smad phosphorylation by attaching to active type I receptors and competing with R-Smads for binding. However, other mechanisms include inhibiting the formation of R-Smad-Smad4 complexes, recruiting Smad-specific E3 ubiquitin protein ligase 1 (Smurf1) and Smurf 2 to promote receptor ubiquitination and degradation, and directly repressing Smad-induced transcriptional responses (Itoh and ten Dijke, 2007).

**FIGURE 1:**
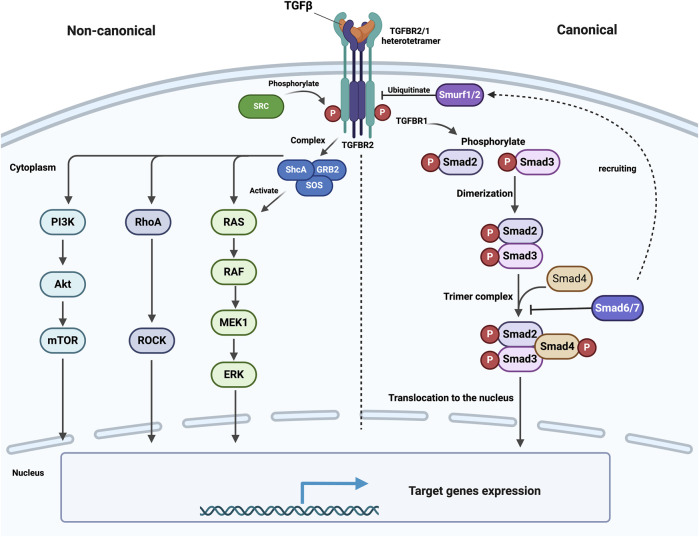
Schematic illustration of canonical and con-canonical TGFβ signaling. Upon ligand binding, TGFBR2 phosphorylates and activates TGFBR1 at specific serine and threonine residues, stabilizing their heterotetrameric structure. Activated TGFBR1 receptors then phosphorylate R-Smads (Smad2 and Smad3), which dimerize and bind to Smad4 to form a trimeric complex. This complex moves to the nucleus and regulates gene expression with other transcription factors. Inhibitory Smads (Smad6 and Smad7) negatively regulate this pathway by preventing R-Smad/Smad4 complex formation and recruiting Smurf1/2, promoting receptor degradation through ubiquitination. On the other hand, TGFBR2 is phosphorylated by SRC, enabling the recruitment of GRB2 and ShcA to activate MAPK signaling. Similarly, TGFBR1 phosphorylates ShcA, forming a ShcA/GRB2/SOS complex that activates Ras, leading to the activation of the MAPK pathway. This figure was generated by Biorender.

While Smad is the primary route by which TGFβ signals, additional pathways, generally known as non-canonical TGFβ signaling, can also be activated by TGFβ and operate alongside the Smad pathway. These signals encompass a range of Mitogen-activated protein kinase (MAPK) pathway, Rho-like GTPase signaling pathway (ROCK), and phosphatidylinositol-3-kinase (PI3K)/AKT/mTOR pathway, as reviewed by (Moustakas and Heldin, 2005; Zhang, 2009). Notably, it should be emphasized that the TGFβ signaling pathway and other pathways can inevitably be involved in crosstalk, as reviewed by Lou, K. (Luo, 2017) (Figure 1).

TGFBR2 can undergo phosphorylation by SRC, a non-receptor tyrosine kinase, which creates a docking site for the recruitment of the SRC homology 2 containing adaptors GRB2 and ShcA. GRB2 forms a complex with SOS, a guanine nucleotide exchange factor for Ras, which in turn connects TGFBR2 to the activation of MAPK (Galliher and Schiemann, 2007; Galliher-Beckley and Schiemann, 2008). Moreover, the creation of a complex between ShcA, GRB2, and SOS can be facilitated by active TGFBR1, which can directly phosphorylate ShcA on tyrosine and serine residues. Afterward, the ShcA/GRB2/SOS complex can activate Ras at the plasma membrane (PM), which in turn activates c-Raf, MEK, and ERK sequentially (Lee et al., 2007) (Figure 1).

## 3 TGFBR1 and TGFBR2: structure and function

**FIGURE 2:**
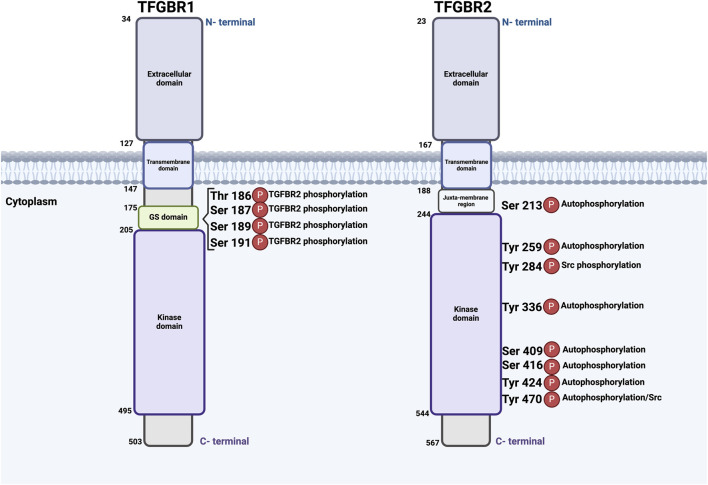
Schematic illustration of TGFBR1 and TGFBR2: Structural Domains and Phosphorylation events. Both receptors possess key functional domains, including extracellular domains, transmembrane domains, and kinase domains. Phosphorylation events occur at different sites; TGFBR2 is autophosphorylated at serine and threonine residues, including Ser213, Ser409, Ser416, Tyr259, Tyr336, Tyr424, and Tyr470, with Tyr284 and Tyr470 being phosphorylated by Src kinase. TGFBR2 phosphorylates TGFBR1 in the GS domain at serine and threonine residues Thr186, Ser187, Ser189, and Ser191. The numbers beside each receptor indicate amino acids. This figure was generated by Biorender.

TGFBR1 and TGFBR2 are structurally and evolutionarily related and share small Cysteine-rich extracellular domains, single transmembrane domains, and intracellular STK domains (Ten Dijke and Arthur, 2007; Hinck, 2012). Furthermore, TGFBR1 includes the highly conserved glycine-serine-rich (GS), which plays a key role in the phosphorylation process. In the GS domain, TGFBR2 phosphorylates TGFBR1 on particular serine and threonine residues (Thr186, Ser187, Ser189, and Ser191) (Wrana et al., 1994; Huse et al., 2001; Chen et al., 2023) (Figure 2).

At the cell surface, TGFBR1 and TGFBR2 were proposed to exist as a mixture of monomeric, homodimeric, and heteromeric complexes, even in the absence of ligands (Zhang et al., 2010; Huang et al., 2011). One common scenario is ligand-induced hetero-oligomerization, where the binding of ligands stabilizes the heterotetrameric form of two TGFBR1 and two TGFBR2 molecules (Ehrlich et al., 2012; Heldin and Moustakas, 2016). Notably, hetero-oligomerization of TGFBR1 and TGFBR2 relies on two regions of each receptor: the cytoplasmic domain of TGFBR1 and a C-terminal region of TGFBR2 (Rechtman et al., 2009). Another scenario suggests that TGFBR1/TGFBR2 may oligomerize in cells as preformed complexes that include both receptor types. Preformed complexes accounted for 25% of receptor complexes on the PM when quantified by the immunofluorescence co-patching assay (Lachmanovich et al., 2003; Ehrlich et al., 2012).

Both TGFBR1 and TGFBR2 undergo posttranslational modifications, such as phosphorylation, ubiquitylation, sumoylation, and N-linked glycosylation, a key modification that ensures proper protein folding, stability, and their trafficking to the cell surface, reviewed by Heldin and Moustakas (Heldin and Moustakas, 2016). Previous findings indicate that the TGFBR2 kinase undergoes complex regulation through autophosphorylation on specific serine and threonine residues, including Ser213, Ser409, and Ser416, Tyr259, Tyr336, Tyr424, and Tyr470 (Luo and Lodish, 1997; Heldin and Moustakas, 2016). Furthermore, Src kinase can phosphorylate TGFBR2 at Tyr284 and Tyr470 (Chen et al., 2014) (Figure 2). Collectively, it becomes clear that TGFBR2 and TGFBR1 undergo both auto-phosphorylation and phosphorylation by cellular kinases. However, the exact kinases and functional importance of this phosphorylation remain unknown (Galliher and Schiemann, 2007).

## 4 Heritable disorders associated with TGFBR1 and TGFBR2

Loss-of-function mutations in TGFBR1 or TGFBR2 have been linked to a range of inherited diseases known as Hereditary Connective Tissue Disorders (HCTDs). They are defined by dysregulated TGFβ signaling and result in diverse clinical manifestations impacting different organ systems, mainly the vascular system (Verstraeten et al., 2017). There are two categories of HCTDs: syndromic forms, such as LDS, MFS2, vEDS, and SGS, and nonsyndromic forms, including TAAD (Lindsay and Dietz, 2011), as well as the rare familial skin cancer MSSE (Goudie, 2020), as described in Table 1. Comprehending the genetic foundation and molecular cellular mechanisms that cause these diseases is crucial for precise diagnosis, evaluating risk, and creating specific treatment strategies. Here, we will delve into these hereditary disorders, emphasizing their clinical characteristics and reported underlying molecular mechanisms and highlighting the gaps in our understanding and future directions.

**TABLE 1:** Monogenic disorders associated with mutations in TGFBR1 and TGFBR2.

Disorder	Phenotypic presentation	Mutation type	Mutation incidence	References
Loeys-Dietz syndrome (LDS)	Generalized arterial tortuosity, hypertelorism, bifid/split uvula, easy bruising of the skin, atrophic scars. (syndromic)	Mainly missense mutations in the STK domain of TGFBR1 or TGFBR2.	The incidence of mutations: TGFBR2, 55%–60%; TGFBR1, 20%–25%; TGFB2, 5%–10%; Smad3, 5%–10%; TGFB3, 1%–5%; and Smad2, 1%–5%.	Loeys et al. (2005), Meester et al. (2017)
Marfan sydrome type 2 (MFS2)	Progressive expansion of the aorta. Prominent aortic, skeletal and skin anomalies. (syndromic)	Mainly missense mutations in the STK domain of TGFBR1 or TGFBR2.	Mutations in TGFBR1 and TGFBR2 are responsible for about 5%–10% of MFS cases.	Mizuguchi et al. (2004), Sakai et al. (2006)
Familial Thoracic aortic aneurysms and dissections (TAAD)	Aortic degeneration, Elastic lamina fragmentation, ECM remodeling, SMCs death, Cystic medial necrosis, Vasa vasorum hemorrhage, Influx of various inflammatory cells. (nonsyndromic)	Missense mutations in the STK domain of TGFBR2.	TGFBR2 mutations are the cause of familial TAAD in 5% of the cases.	Pannu et al. (2005), Albornoz et al. (2006)
Multiple self-healing squamous epithelioma (MSSE)	Numerous skin tumors on the face and limbs. (nonsyndromic)	Loss-of-function mutations, including nonsense and frameshift variants clustered in the STK domain or the extracellular ligand-binding domain of TGFBR1.	Primarily caused by loss-of-function mutations in TGFBR1 combine with permissive variants at a second related locus on the long arm of chromosome 9.	Goudie et al. (2011), Goudie (2020)
Vascular Ehlers-Danlos syndrome (vEDS)	spontaneous rupture of the intestine or other intra-abdominal organs, as well as aneurysms or ruptures of main arteries. (syndromic)	Missense mutations in STK domain of TGFBR1 and TGFBR2.	9.6% of likely pathogenic variants in various genes including TGFBR1 and TGFBR2.	Weerakkody et al. (2016), Renner et al. (2019)
Shprintzen-Goldberg syndrome (SGS)	Nearly all of the craniofacial, skeletal, cutaneous, and cardiovascular features of MFS and LDS, along with mental impairment and severe skeletal muscle hypotonia. (syndromic)	Missense mutation in TGFBR2.	Studies are limited.	Robinson et al. (2005), Van Steensel et al. (2008)

### 4.1 Loeys-Dietz syndrome (LDS)

In 2005, LDS was initially identified as an autosomal dominant inherited connective tissue disorder. Although there is considerable clinical overlap between LDS and MFS syndrome, the vascular and skeletal features of LDS distinguish it from MFS. Typical LDS findings include generalized arterial tortuosity, hypertelorism, bifid/split uvula, easy bruising of the skin, or atrophic scars (Loeys et al., 2005; Loeys et al., 2006). Individuals diagnosed with LDS exhibit a more aggressive and detrimental cardiovascular risk profile and a higher likelihood of developing aortic dissection at a younger age and smaller vascular dimensions (Loeys et al., 2005; Loeys et al., 2006).

The genetic cause of LDS was initially identified as loss-of-function mutations in *TGFBR1* (LDS type 1, OMIM# 609192) and *TGFBR2* (LDS type 2, OMIM# 610168), which disrupt receptor-ligand interactions and downstream signaling transduction, resulting in impaired TGFβ signaling (Loeys et al., 2005). Subsequently, it has been found that mutations in other TGFβ signaling components can also lead to LDS phenotype and perturb the signaling dynamics, which include mutations in *SMAD3* (LDS type 3, OMIM# 613795), *TGFB2* (LDS type 4, OMIM# 614816) and *TGFB3* (LDS type 5, OMIM# 615582) (van de Laar et al., 2011; Lindsay et al., 2012; Bertoli-Avella et al., 2015; Schepers et al., 2018). There are no observable differences in phenotypic characteristics between patients with mutations in *TGFBR1* and *TGFBR2*, and no evident phenotype-genotype correlations have been evaluated so far (Pezzini et al., 2012). However, the incidence of mutations in *TGFBR2* (55%-60%) is higher than that in other genes (*TGFBR1*, 20%–25%; *TGFB2*, 5%–10%; *SMAD3*, 5%–10%; *TGFB3*, 1%-5%; and *SMAD2*, 1%-5%) (Meester et al., 2017). Most mutations in TGFBR1 and TGFBR2 in LDS are missense and distributed across various functional domains of these receptors and principally in the STK domain of either receptor (Loeys et al., 2006; Sakai et al., 2006; Stheneur et al., 2008).

Mutant receptors in cells lacking TGFBRs were shown to be unable to sustain TGFβ signaling. Reduced TGFβ signaling was taken for granted as a detrimental mechanism in the disease, and the mutant receptor subunits could not cycle or traffic to the cell surface (Mizuguchi et al., 2004). In addition, it was anticipated that most vascular phenotype-associated TGFBRs mutations would result in a mutant receptor protein capable of surface trafficking and extracellular ligand binding but defective in its ability to transmit the intracellular signal (Mizuguchi et al., 2004; Pezzini et al., 2012). Additionally, one defective receptor molecule might completely deactivate the TGFβ receptor complex, which consists of two molecules of each TGFBR1 and TGFBR2. Nevertheless, studies that combined the WT and mutant receptors showed that there was a slight reduction in TGFβ signaling, with a signal loss of no more than 50%. This indicates that the combination of WT and defective TGFBR1 or TGFBR2 may still transmit signals (Mizuguchi et al., 2004; Cardoso et al., 2012).

On the other hand, it is hard to square the strong evidence that several aspects of LDS, including those that overlap with MFS, are caused by excessive TGFβ signaling and may be reduced or avoided by TGFβ antagonists in animal models, with a paradigm that just invokes reduced TGFβ signaling. Moreover, Loeys et al.‘s findings revealed a paradoxical increase in TGFβ signaling activity in LDS patient aortic tissues (Loeys et al., 2005). This enhanced signaling is evidenced by the upregulation of TGFβ target gene expression and heightened downstream signaling in aortic walls and aortic cell cultures isolated from LDS patients, including collagen, connective tissue growth factor, and phosphorylated Smad2. All are suggestive of increased TGFβ signaling activity, which is implicated in the pathogenesis of LDS’s diverse clinical manifestations (Loeys et al., 2005; Maleszewski et al., 2009).

Maleszewski et al. reported mutations in *TGFBR1* and *TGFBR2* that lead to the abnormal activation of the TGFβ signaling pathway. This dysregulation results in excessive or inappropriate signaling that contributes to the structural abnormalities observed in the aortic wall of LDS patients (Maleszewski et al., 2009). The increase in TGFβ signaling is associated with several key histopathologic changes, such as extensive medial degeneration, including smooth muscle cell (SMC) loss and elastic fiber fragmentation. These changes are thought to be driven by the overactive TGFβ signaling pathway, which promotes the abnormal remodeling of the extracellular matrix. An important observation from the study, as well, was the increased nuclear accumulation of phosphorylated Smad2 (pSmad2) in the aortic walls of individuals with either LDS or MFS, suggesting enhanced activation of the canonical pathway (Maleszewski et al., 2009; Pezzini et al., 2012).

This paradoxical increase in TGFβ signaling observed in LDS may be explained by the differential activation of canonical and non-canonical pathways through distinct functional mechanisms of the receptor complex. It has been proposed that specific kinase activities are responsible for the phosphorylation of Smad proteins and the adaptor protein ShcA, which is critical for initiating the ERK signaling cascade (Lee et al., 2007). Furthermore, receptor-mediated ubiquitination involving the ubiquitin ligase TRAF6 has been linked to the activation of other MAPKs by TGFβ (Yamashita et al., 2008). In the context of LDS, if abnormal receptor complexes selectively maintain non-canonical signaling, while canonical signaling predominantly governs feedback regulation, compensatory mechanisms such as elevated ligand expression or activation could result in an increase in non-canonical TGFβ signaling in a cell-autonomous manner (Lindsay and Dietz, 2011).

It is important to note that the three TGFβ ligands, TGFB1, TGFB2, and TGFB3, have distinct functions and tissue-specific roles. TGFB2 and TGFB3 are shown to have important, yet more specific, roles in tissue remodeling, fibrosis, and vascular pathology, particularly in MFS and LDS, where their variants are strongly linked to the development of aortic aneurysms. In contrast, TGFB1, while crucial in other tissue processes such as fibrosis, does not appear to have the same level of significance in vascular pathology (Lichtman et al., 2016; Deleeuw et al., 2023).

### 4.2 Marfan syndrome type 2 (MFS2)

Classical MFS (OMIM 154700) is a variable autosomal dominant HCTD. It has significant effects on the cardiovascular, ocular, skeletal, and other organ systems. The birth incidence is roughly 1 in 3,000–5,000 individuals (Salik and Rawla, 2019). Symptoms include progressive expansion of the aorta, typically at the sinus of Valsalva, which is often accompanied by aortic valve leakage. This can result in aortic dissection or rupture, which is the leading cause of mortality in MFS cases (Gray et al., 1998; Salik and Rawla, 2019).

In 1991, mutations in the *FBN1* gene at 15q21.1, which codes for fibrillin-1, a primary component of the extracellular matrix (ECM) microfibrils, were identified as the cause of MFS. These mutations can affect the structure, stability, or function of fibrillin-1 and its interactions with other ECM components (Collod-Béroud and Boileau, 2002; Ramirez and Dietz, 2007). In addition, fibrillin-1, along with other microfibrils, plays a crucial role in regulating the bioavailability and function of TGFβ ligands. Those ligands are produced primarily in an inactive state, known as a large latent complex, which includes the ligand, latency-associated peptide, and latent TGFβ binding protein, all anchored to ECM by fibrillin-1 (Jensen et al., 2012). In a typical scenario, microfibril degradation occurs as a result of particular physiological stimuli or enzymatic proteolysis, which allows for the release of diffusible active TGFβ. However, one hypothesis stated that impaired or decreased expression of fibrillin-1 in MFS hinders TGFβ sequestration, which in turn leads to overactivity of TGFβ signaling cascades, a key factor in the pathophysiology of MFS (Neptune et al., 2003; Lindsay and Dietz, 2011).

Later on in 2004, a Japanese family manifesting MFS clinical symptoms was found to have a complex *de novo* chromosomal rearrangement and carrying a 3p24.1 chromosomal breakpoint, with no evidence of an *FBN1* mutation. *In-vitro* studies discovered that the chromosomal breakpoint only impacted the *TGFBR2* gene, which was eventually identified as MFS2 (OMIM 154705) (Mizuguchi et al., 2004). This finding provided the initial genetic evidence establishing a direct connection between aberrant TGFβ signaling and HCTDs in humans. Moreover, mapping genes at 3p24.2-p25 in a large French family prompted an evaluation of TGFBR2 association with MFS. In addition, ten Japanese patients and nine French probands with MFS were examined; none of these patients exhibited a mutation in *FBN1*. Three missense mutations in TGFBR2 were found: 923T>C (L308P), 1346C>T (S449F), and 1609C>T (R537C). Notably, all the missense mutations were located at a highly conserved STK domain of TGFBR2 (Mizuguchi et al., 2004; Boileau et al., 2005).

Subsequently, another study examined the genetic analysis of 49 patients exhibiting symptoms corresponding to MFS. In the absence of *FBN1* mutations, other mutations affecting *TGFBR1* and *TGFBR2* were identified. Collectively, it was concluded that the majority of MFS cases were shown to be caused by *FBN1* mutations, whereas mutations in *TGFBRs* were responsible for about 5%–10% of the cases (Sakai et al., 2006).

The initial dominating idea of the pathology of MFS was the decreased TGFβ signaling as demonstrated by the study of Mizuguchi et al., which elucidates the pathogenic mechanism by which heterozygous mutations in the *TGFBR2* gene, mainly in the STK domain and affecting highly conserved amino acids, lead to MFS through dysregulated TGFβ signaling (Mizuguchi et al., 2004). Interestingly, even with loss-of-function mutations in TGFBRs, there is evidence of upregulation of TGFβ signaling in the aortic walls of MFS2-affected individuals. This suggests complex regulatory mechanisms that lead to increased signaling activity despite receptor dysfunction (Takeda et al., 2018). This suggests that complex regulatory mechanisms may enhance downstream signaling activity even in the presence of dysfunctional receptors. Notably, this apparent signaling increase may not be a direct consequence of the mutations themselves, but rather a secondary response, potentially linked to compensatory mechanisms or wound-healing processes triggered by structural damage in the aorta (Marcos-Ríos et al., 2025), as will be discussed further.

### 4.3 Familial thoracic aortic aneurysms and dissections (TAAD)

TAAD are characterized histologically by degeneration of the entire aortic wall (intima, media, and adventitial layer), involving elastic lamina fragmentation, ECM remodeling, SMCs death, cystic medial necrosis, vasa vasorum hemorrhage, and influx of various inflammatory cells (Albornoz et al., 2006; Goldfinger et al., 2014). Additionally, it has been shown that familial TAAD tends to cluster among families since over 20% of individuals with a TAAD who do not have a documented vascular HCTD have a first-order family relation who has had an aortic aneurysm (Coady et al., 1999; Albornoz et al., 2006).

There are a minimum of 29 genes that have been linked to TAAD development; most of these genes encode proteins that are involved in the ECM, SMC contraction or metabolism, or the TGFβ signaling pathway. The vast majority of them have an autosomal dominant inheritance pattern characterized by low penetrance and variable expression (Brownstein et al., 2017). In 2005, Pannu et al. discovered that germline *TGFBR2* mutations are the cause of the inherited predisposition to familial TAAD in five percent of the cases. A total of four unrelated TAAD families who had *TGFBR2* mutations reported mutations affecting arginine 460 in the receptor’s cytoplasmic STK domain. Although the ascending aortic disease was the most common cardiovascular manifestation in families with *TGFBR2* mutations, members of these families also experienced substantial descending aortic disease and aneurysms of other arteries (Pannu et al., 2005).

### 4.4 Multiple self-healing squamous epithelioma (MSSE)

MSSE (OMIM# 132800) is a rare familial autosomal dominant skin cancer that presents as numerous skin tumors on the face and limbs. These lesions naturally heal over time, but if they are not removed, they can leave behind distinctive pitted scars. Such a condition emerges when loss-of-function mutations of *TGFBR1* combine with permissive variants at a second related locus on the long arm of chromosome 9 (Goudie, 2020). Further, Microdissected tumor DNA from MSSE patients with constitutional *TGFBR1* mutations reveals somatic loss of heterozygosity at the *TGFBR1* locus, with the mutant allele retained, suggesting that *TGFBR1* acts as a tumor suppressor gene (Bose et al., 2006). It appears that tumor development is unaffected by constitutional heterozygous loss-of-function *TGFBR1* mutations, indicating that the majority of cells with a single functioning copy of the *TGFBR1* gene have not experienced significant disruptions in TGFβ signaling (Goudie et al., 2011; Goudie, 2020). In comparison, skin keratinocytes may transform into tumors if a “second hit” causes them to lose the one functioning *TGFBR1* allele.

Previously discussed MFS-related syndromes have also been linked to heterozygous loss-of-function mutations of *TGFBR1,* including MFS2, LDS, and TAAD. Interestingly, while MSSE predisposes to skin cancer, these vascular conditions do not appear to confer cancer risk. This phenotypic divergence is thought to result from the distinct mutational spectra observed in *TGFBR1*. MSSE is primarily caused by loss-of-function mutations, including nonsense and frameshift variants clustered in the cytoplasmic STK domain or the extracellular ligand-binding domain. By contrast, MFS-related *TGFBR1* mutations are not truncating, but rather consist mostly of missense mutations or in-frame deletions/duplications. These variants are hypothesized to result in altered signaling, particularly enhanced TGFβ pathway activation, which contributes to vascular pathology (Goudie et al., 2011; Goudie, 2020).

### 4.5 Ehlers-Danlos syndrome (EDS)

The EDS encompasses a cluster of related HCTDs (Beighton et al., 1998). The most common symptoms seen in clinical trials were hypermobility of the joints and frequently bruised and fragile skin. There is a varying degree of consequences for internal organs and blood vessels due to ubiquitous connective-tissue fragility (De Paepe and Malfait, 2012). Sudden spontaneous rupture of the intestine or other intra-abdominal organs, as well as aneurysms or ruptures of main arteries, can manifest in vascular EDS individuals as early as the first 2 weeks of birth (Pepin et al., 2000).

The clinical classification of EDS was established by Villefranche nosology in 1997, (Beighton et al., 1998). Three main categories of EDS exist: classical, vascular, and hypermobility-type. Collagen I, III, and V are known to be affected by certain microscopic, biochemical, and genetic abnormalities; these dysfunctions impact the packing and stability of collagen fibrils forming an ECM network, which is the primary pathogenic factor. The majority of classical EDS cases are caused by mutations in the *COL5A1* or *COL5A2* genes, which encode type V collagen; vascular EDS is mainly caused by mutations in the *COL3A1* gene, which encodes type III collagen; and the genetics of hypermobility-type EDS is estimated to be diverse and not fully clear (Pepin et al., 2000; De Paepe and Malfait, 2012). Later, next-generation sequencing (NGS) was used to sequence a panel of relevant collagen and aortopathy genes in EDS patients; the aortopathy NGS panel uncovered four novel variants in *FBN1*, *TGFBR1*, and *TGFBR2* that have eluded clinical and genetic investigations to date. Of these, three were thought to be potentially pathogenic; two were located in the STK domain of TGFBR1 and TGFBR2, which are linked to most variants of LDS, and the third was in the vicinity of Smad3’s MH2 domain (Weerakkody et al., 2016).

A recent study utilized NGS gene testing methods in a cohort of 199 patients who have hereditary arthropathies, they detected one pathogenic variation in either *FBN1* or *SMAD3* in 15 patients (7.5%) and at least one likely pathogenic variant in 19 patients (9.6%), including thirteen novel pathogenic/likely pathogenic variants in various genes including *TGFBR1* and *TGFBR2* (Renner et al., 2019).

### 4.6 Shprintzen-Goldberg syndrome (SGS)

SGS (OMIM# 182212) is a systemic HCTD that contains nearly all of the craniofacial, skeletal, cutaneous, and cardiovascular features of MFS and LDS, along with mental impairment and severe skeletal muscle hypotonia (Robinson et al., 2005). Enhanced activation of TGFβ signaling and higher expression of TGFβ responsive genes were observed in cultured dermal fibroblasts from SGS patients. Additionally, it was found that a variant in the proto-oncogene Sloan Kettering Institute (*SKI*) is known to suppress TGFβ activity (Doyle et al., 2012). Members of the SKI family have a role in inhibiting Smad-dependent TGFβ signaling. They accomplish that by blocking the activation of Smad2/3, hindering the translocation of activated R-Smad/Co-Smad complexes into the nucleus, and reducing the expression of TGFβ target genes (Prunier et al., 2003). Further, it was discovered that the *SKI* gene has a dominantly inherited heterozygous in-frame deletion in exon 1, which was detected by family-based exome sequencing. The R-Smad binding domain of SKI was the exclusive site of all mutations found in exon 1 (Carmignac et al., 2012).

The causal connection of SGS to TGFBRs was also detected in a patient having *de novo* heterozygous *TGFBR2* splicing defect IVS5-2A > G, leading to a 10-amino acid insertion in the STK domain (Kosaki et al., 2006). Further, a clinically diagnosed SGS patient was reported to have a novel missense mutation in *TGFBR2*, which caused a lysine to substitute a threonine at position 516 in the STK domain (Van Steensel et al., 2008). Further genotype-phenotype correlation studies are still to be elucidated.

## 5 Molecular mechanisms of diseases associated with TGFBR1 and TGFBR2

**FIGURE 3:**
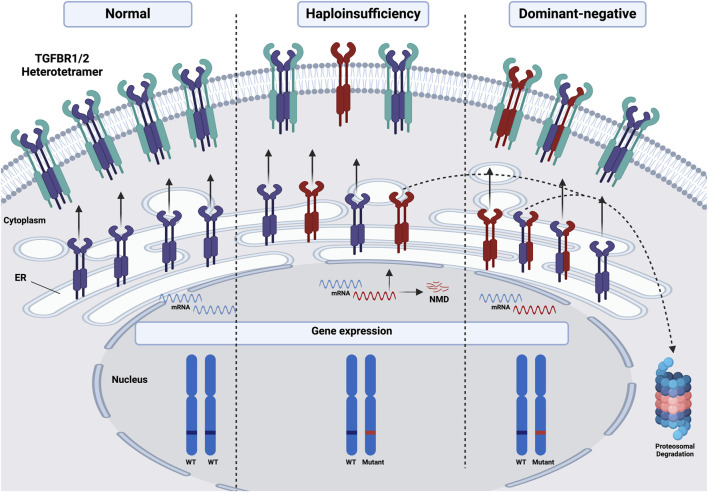
Schematic illustration of the molecular mechanisms underlying diseases associated with mutations in *TGFBR1* and *TGFBR2*. Under normal conditions, both alleles of either receptor in a diploid locus are expressed and trafficked to the PM, where they form functional heterotetramers with the other receptor. However, haploinsufficiency occurs when one allele is mutated or deleted, leading to either nonsense-mediated mRNA decay (NMD) of the mutated allele or the proteasomal degradation of the mutant receptor protein, or the trafficking of a non-functional mutant protein to the PM, resulting in a reduction of functional protein into half compared to WT. Dominant-negative effects can further exacerbate HI, as mutant receptor proteins may interfere with WT proteins, either promoting their retention in the ER followed by proteasomal degradation or hindering the formation and function of heterotetramers at the PM, further diminishing overall receptor activity and signaling output. Red: mutated, Purple/Green: either TGFBR1 or 2. This figure was generated by Biorender.

To date, the molecular mechanisms underlying diseases associated with mutations in *TGFBR1* and *TGFBR2* have been linked to haploinsufficiency (HI) and, in some cases, dominant-negative effects. These two fundamental mechanisms can drive the onset and course of various genetic disorders, particularly autosomal dominant diseases in which one allele expresses the mutant protein while the other allele maintains its WT expression (Seidman and Seidman, 2002; Gariballa et al., 2024b). HI occurs when a typically diploid locus has one mutated or deleted allele, and the remaining functional allele fails to produce sufficient protein to support normal physiological functions (Veitia, 2002). In contrast, dominant-negative effects are caused by mutant proteins that not only lack their normal function but actively interfere with their WT counterparts, often forming non-functional complexes that can bind to and inactivate the normal protein, reducing overall functional protein activity even if one gene copy is intact (Veitia, 2007), as shown in Figure 3. Additionally, we are proposing that some mutations could interfere with receptors trafficking to the cell membrane, resulting in their retention in the ER and subsequent degradation *via* the ERAD pathway, as will be discussed further.

### 5.1 Haploinsufficiency (HI)

HI plays a significant role in various autosomal dominant diseases. For example, Horbelt et al. highlight that some mutations in *TGFBR2* lead to a reduced gene dosage effect, which is characteristic of HI. The study found that the severity of clinical manifestations in patients correlates with the level of impairment in Smad signaling. When TGFBR2 mutations reduce the receptor’s ability to activate Smad proteins, it results in insufficient signaling, leading to connective tissue defects and other symptoms typical of MFS and related disorders such as LDS (Horbelt et al., 2010). Moreover, Fujiwara et al. revealed that TGFBR1 variants can cause disease through mechanisms that include both HI and a dominant-negative effect. HI arises when one allele of *TGFBR1* is mutated, leading to reduced signaling activity due to decreased protein levels. This under-activity in the TGFβ pathway is sufficient to disrupt normal cellular functions, contributing to the development of systemic features in LDS, such as aortic aneurysms and other connective tissue phenotypes. In contrast, a splice donor site variation in intron 5 (c.973 + 1 G>A) in a familial case of LDS was defined. It is anticipated that this mutation will have a dominant-negative effect by causing the in-frame deletion of exon five within the STK domain (Fujiwara et al., 2018; Fujiwara et al., 2019).

### 5.2 Defective receptor trafficking and dominant-negative effects

Once inside the ER, unfolded secretory and endomembrane proteins in eukaryotic cells begin the crucial initial steps in achieving their correct tertiary conformations (Liu and Ye, 2011). Cells have developed vast ERQCM systems to ensure that only correctly folded proteins reach their functional destination effectively and reliably (Sun and Brodsky, 2019).

Mutations can lead to the production of proteins that are structurally abnormal and prone to misfolding. These misfolded proteins are recognized by the cellular quality control mechanisms and are retained within the ER and subsequently degraded through the ERAD, thus preventing their transport to their intended destinations on the cell surface. The ER retention mechanism is a protective response to prevent malfunctioning proteins from disrupting cellular processes. However, this retention can lead to an accumulation of misfolded proteins within the ER, triggering ER stress and activating the unfolded protein response. Prolonged ER stress and chronic activation of the unfolded protein response can lead to cellular dysfunction, apoptosis, and contribute to disease pathology (Hetz, 2012; Duttler et al., 2013). Further, around 12%–15% of newly synthesized proteins fail to reach their desired conformation and are subsequently eliminated through ERAD, this proportion usually rises dramatically when proteins have mutations that cause them to miss- or mal-fold (Guerriero and Brodsky, 2012; Oikonomou and Hendershot, 2020; Chen et al., 2023).

It is essential to note that the ERAD pathway is integral to cellular homeostasis, particularly in the context of hereditary monogenic disorders involving the TGFβ signaling pathway. Our previous work has highlighted the implication of ERAD in the loss of function mutations in some TGFβ receptors such as endoglin, Activin Receptor-Like Kinase-1 (ALK1), and Bone morphogenetic protein receptor (BMPR2), which cause Hereditary Haemorrhagic Telangiectasia (HHT1 and HHT2) and familial pulmonary arterial hypertension, respectively (Ali et al., 2011; Hume et al., 2013; Gariballa et al., 2022). Mutations in these genes can lead to the production of misfolded variants, and ERAD is responsible for identifying and degrading misfolded proteins within the ER. While this degradation is crucial for reducing ER stress, it can also result in decreased receptor availability on the cell surface, impairing TGFβ signaling. The extent of ERAD activity, therefore, directly influences the severity of these disorders, as excessive degradation can exacerbate symptoms by further diminishing the already compromised signaling capacity (Gariballa and Ali, 2020).

## 6 Is there a role for the dominant-negative effect in the pathogenesis of some *TGFBR1* and *TGFBR2* disease-causing variants?

In autosomal dominant diseases, the dominant-negative effect aggravates the situation by not only reducing the function of the remaining normal protein but also actively interfering with its activity, contributing to a more severe disease phenotype (Lindsay and Dietz, 2011; Gerasimavicius et al., 2022).

Dominant-negative effects are widely implicated in the molecular mechanisms of several autosomal dominant diseases, including those involving mutations in the TGFβ signaling pathway. This phenomenon is particularly evident in HHT1, where some mutations in the gene encoding endoglin have been shown to disrupt normal receptor function. We have recently provided significant insights into how these dominant-negative effects contribute to disease mechanisms and are likely to exacerbate the associated phenotypes. Mutant endoglin proteins often misfold and are retained within the ER. These ER-retained misfolded proteins can interact with the normal WT counterpart in early biogenesis in the ER and form WT/mutant mixed dimers that impede the normal trafficking of a significant fraction of WT endoglin, ultimately leading to a compound loss of function (Hume et al., 2013; Gariballa et al., 2024a; Gariballa et al., 2024b). This scenario results in a double hit to receptor functionality: the mutant proteins reduce the number of functional receptor complexes available at the cell surface, and the ERAD pathway further diminishes receptor availability by degrading normal proteins (Gariballa et al., 2024b). Furthermore, ALK1 variants linked to HHT2 result in misfolded proteins that accumulate in the ER. These variants are proposed to exert a dominant-negative effect on the WT protein by forming non-functional heterodimers at the plasma membrane (Hume et al., 2013; Jain et al., 2023).

Concerning the dominant-negative effect of mutations in *TGFBR1* and *TGFBR2*, to our knowledge, only two studies had reported the implication of dominant-negative effects of the mutant variants of these genes on the WT counterpart expressed by the functional allele. Horbelt et al. demonstrated that specific mutations in the *TGFBR2* gene, R528C, R528H, R537C, R537P, and R460H, not only lead to a loss of normal receptor function but also exert dominant-negative effects, interfering with the activity of the remaining WT receptors in MFS2 and LDS. The analysis also reveals that the extent of Smad/ERK signaling activity correlates with the severity of phenotypic outcomes in MFS and related disorders (Horbelt et al., 2010). In addition, Cardoso et al. demonstrated that some mutations in *TGFBR1*, such as K232R and R487P, are not merely inactivating but also exert a dominant-negative impact on the function of the receptor in LDS (Cardoso et al., 2012).

Here, we hypothesize that the ERAD mechanism compounded by dominant-negative effects could be implicated in the pathogenesis of diseases associated with some TGFBR1 and TGFBR2 mutations. Given that ERAD plays a crucial role in identifying and degrading misfolded proteins, it is plausible that mutations in TGFBR1 and TGFBR2 lead to the production of misfolded receptor variants, which are then retained in the ER and targeted for degradation. This excessive degradation could reduce receptor availability on the cell surface, thereby impairing TGFβ signaling pathways and contributing to the development of associated phenotypes. Exploring the possible involvement of ERAD in these disorders and elucidating the underlying mechanisms of receptor dysfunction will not only provide insight into the pathogenesis of these disorders but also open avenues for novel therapeutic strategies.

## 7 Could the phenotypic variability of diseases associated with TGFBR1 and TGFBR2 be explained by the interplay between these entwined complex mechanisms?

Past research has shown that individuals with familial TAAD demonstrated an autosomal dominant pattern of inheritance, with most individuals experiencing issues with the ascending thoracic aorta (Hasham et al., 2003). The missense mutation located in the STK domain of TGFBR2, p. Arg460Cys (c.1378C>T), prevents the catalytic loop from maintaining its structural integrity and hinders efficient signaling (Pannu et al., 2005). Following that, the mutation p. Arg460His (c.1379G>A) was identified in a familial MFS2 case characterized by skeletal and cardiovascular symptoms without significant ocular manifestations (Disabella et al., 2006), evidence of various phenotypes resulting from mutations at the same position, p. Arg460.

Similarly, Mutations in TGFBR1 could cause LDS and MSSE, two medical conditions that are clinically distinct. A recent study showed that the variants (c.973+1G>A and c.806-2A>C) of TGFBR1 cause both LDS and MSSE, respectively. Their results of the *ex-vivo* minigene splicing assay support their hypothesis that missense variants in STK domains induce LDS, while splice site mutations in STK domains induce MSSE by activating two distinct cryptic splice sites and resulting in in-frame and out-of-frame transcripts, respectively (Fujiwara et al., 2019). However, further investigation is required to understand the mechanisms by which variants in *TGFBR1* give rise to two clinically distinguished diseases.

More precisely, individuals who carry an identical missense mutation in *TGFBR2* may have varying degrees of disease severity in LDS. In particular, certain individuals may exhibit severe cardiovascular symptoms like aortic aneurysms, whereas others may experience significantly milder symptoms or even no symptoms at all (Loeys et al., 2006). The variant TGFBR2 c.1067G > C (p. Arg356Pro) was found in patients exhibiting classic symptoms of LDS, including descending pseudoaneurysm, bilateral carotid tortuosity, bifid uvula, and hypertelorism (CS, 2005). Surprisingly, a later study discovered that one Chinese father of an LDS patient harboring the same variant was healthy despite having the same genetic mutation. After closely analyzing the father’s cardiac structure and arterial tree, no obvious abnormalities were found, except for a minor decrease in left ventricular diastolic function (Yang et al., 2020).

The observed variation in phenotypes may be attributable to variations in the residual functionality of the mutant TGFBR2 or TGFBR1 protein: the existence of genetic modifiers, epigenetic modification, interplay with other signaling pathways, or environmental factors.

Despite significant advances in identifying mutations and genes associated with HCTDs, critical gaps remain in understanding the clinical and biological consequences of these mutations. Most importantly, the literature does not yet support a clear or consistent genotype–phenotype correlation. Several layers of complexity are likely to contribute to this challenge. First, there is a lack of comprehensive structural analyses, such as molecular dynamics simulations and stability prediction data, to assess the impact of specific mutations. For instance, the TGFBR2 R460C mutation, reported in TAAD patients, was studied by Pannu et al., who used homology modeling to show that substitutions at R460 disrupt F-helix–D-helix communication, leading to reduced signaling capacity of the receptor (Pannu et al., 2005).

To illustrate the structural and functional complexity of TGFBR2 mutations, we highlight the case of two distinct missense variants affecting the same residue, R537C and R537P, which are associated with MFS2 and LDS, respectively (Mizuguchi et al., 2004; Horbelt et al., 2010). Although both substitutions disrupt the native arginine at position 537, they exert distinct biophysical effects. Arginine at this site likely contributes to stabilizing the α-helix through ionic interactions or hydrogen bonding. Replacement with cysteine (R537C) may lead to loss of positive charge and disruption of salt bridges, potentially resulting in a moderately destabilized structure with partial retention of function. In contrast, substitution with proline (R537P) introduces a rigid cyclic side chain known to induce kinks in α-helices, which is predicted to severely disrupt local secondary structure. This stark difference in structural impact may explain the phenotypic divergence observed between MFS2 and LDS patients, despite the identical residue being affected, highlighting the need for further structural studies. Similar structure-function relationships have been described in other TGFβ signaling components; for instance, in TGFB3, substitutions such as p. Arg300Trp or p. Leu401Pro were shown to disrupt key ionic and hydrophobic interactions, leading to impaired protein stability and receptor binding (Bertoli-Avella et al., 2015). Second, there is a shortage of well-curated patient cohorts with detailed and standardized clinical characterization, which limits the ability to systematically correlate genotypes with phenotypes. Collaborative studies with international registries or biobanks for large and clinically well-annotated cohorts are essential for meaningful validation of variant pathogenicity across diverse populations. Finally, there remains an urgent need to elucidate the diverse molecular and cellular mechanisms through which different mutations exert their effects. These may include haploinsufficiency, hypomorphic function, ER-associated degradation, or dominant-negative effects, as discussed earlier in our review. Such mechanistic diversity further complicates efforts to establish direct genotype-phenotype correlations. For example, Horbelt et al. reported that variants such as R528C and R537C exert dominant-negative effects, markedly impairing Smad signaling activity. In contrast, the R460C mutation, linked primarily to TAAD, exhibited a milder dominant-negative effect and preserved partial Smad phosphorylation and transcriptional response (Horbelt et al., 2010). These observations suggest a correlation between the extent of signaling disruption and the clinical severity of the associated connective tissue disorder, highlighting the need to consider all structural, functional, and clinical data when interpreting the pathogenic impact of individual variants.

## 8 Mitochondrial dysfunction and ECM-TGFβ crosstalk in TGFBR-associated disorders

An emerging dimension in the pathogenesis of TGFBR-associated HCTDs is the interplay between the ECM and mitochondrial function. It has become evident that cells are highly sensitive to alterations in the ECM, and such changes can initiate a wound–healing–like response characterized by localized hypoxia and a shift in cellular metabolism (Zhang et al., 2024). This response involves a downregulation of mitochondrial oxidative phosphorylation and a compensatory upregulation of anaerobic glycolysis, resembling the metabolic adaptation seen in wound healing and cellular stress responses. While this response is beneficial in acute repair, in chronic connective tissue disorders such as MFS and LDS, and related disorders, persistent ECM remodeling may lead to sustained mitochondrial suppression, contributing to chronic inflammation and fibrosis (Zhang et al., 2024; Marcos-Ríos et al., 2025).

Persistent suppression of mitochondrial activity has been shown to result in mitochondrial dysfunction, increased reactive oxygen species, impaired ATP production, and eventual cellular exhaustion. This has been well-documented in aortic aneurysm tissues and vascular SMCs derived from MFS models (Gäbel et al., 2021; Oller et al., 2021; Verhagen et al., 2021; Oller et al., 2022). These metabolic abnormalities are not merely a consequence of tissue damage but appear to be integral to disease progression. In particular, TGFβ signaling has emerged as a central mediator of this response, linking ECM sensing to metabolic reprogramming. Importantly, TGFBR2-mutant cells have also been shown to exhibit pronounced mitochondrial dysfunction, further supporting the hypothesis that TGFβ-driven ECM remodeling and mitochondrial impairment are interconnected pathogenic mechanisms in these disorders (Van Der Pluijm et al., 2018). Moreover, TGFβ activation in this context may not solely represent a primary pathogenic driver but also reflect a secondary wound healing response to ongoing tissue damage (Penn et al., 2012). Thus, any individual differences in this wound healing response in related genes may influence the overall pathological phenotype of the TGFBR mutations.

## 9 Conclusions and future perspectives

In conclusion, the intricate role of TGFBR1 and TGFBR2 in the TGFβ signaling pathway underscores their significance in maintaining cellular and tissue homeostasis. Mutations in these receptors are implicated in a spectrum of monogenic disorders, highlighting the pathway’s critical involvement in connective tissue integrity and cardiovascular function. The diverse clinical manifestations observed in conditions including LDS, MFS2, TAAD, MSSE, vEDS, and SGS illustrate how genetic variations can lead to distinct and overlapping phenotypes despite affecting the same genes. In addition, the complexity of TGFβ signaling dysregulation is further compounded by the involvement of other signaling pathways, including the PI3K/AKT/mTOR, ROCK, and MAPK, and the variable effect of mutations (hypomorphicity).

In autosomal dominant diseases, HI and dominant-negative effects significantly influence disease severity. Mutations in *TGFBR1* and *TGFBR2* contribute to MFS and related disorders through HI, dominant-negative effects, and possibly other mechanisms that are yet to be investigated, disrupting TGFβ signaling. Our understanding of ERAD’s impact on receptor availability and signaling capacity reveals an additional layer of complexity in disease severity and variability, emphasizing the need for deeper exploration into how ERAD influences disease outcomes.

Investigating the cellular mechanisms of hereditary monogenic diseases associated with TGFBR1 and TGFBR2 enriches our understanding of the pathogenesis and variability of these disorders. Further research is required to examine the impact of these mutations and to clarify the precise molecular mechanisms involved. This deeper insight is vital not only for grasping the fundamental molecular pathology but also for informing the development of targeted therapeutic strategies.

## Glossary

ACVR2A: Activin A Receptor Type 2A
ACVR2B: Activin A Receptor Type 2B
ALK5: Activin Receptor-Like Kinases 5
AMHR2: Anti-Müllerian hormone receptor type 2
BMPR2: Bone morphogenetic protein receptor Type 2
ECM: Extracellular matrix
ER: Endoplasmic reticulum
ERAD: Endoplasmic reticulum-associated degradation
ERK: Extracellular signal-regulated kinase
ERQCM: ER quality control mechanisms
GRB2: SRC homology 2–containing adaptors
GS domain: Glycine-serine-rich domain
HCTD: Hereditary Connective Tissue Disorders
HHT1: Hereditary Haemorrhagic Telangiectasia type 1
HHT2: Hereditary Haemorrhagic Telangiectasia type 2
HI: Haploinsufficiency
I-SMAD: Inhibitory Smad
LDS: Loeys-Dietz syndrome
MAPK: Mitogen-activated protein kinase pathway
MFS: Marfan syndrome
MFS2: Marfan syndrome type 2
MSSE: Multiple Self-healing Squamous Epithelioma
NGS: Next-generation sequencing
PI3K/AKT: phosphatidylinositol-3-kinase pathway
PM: Plasma membrane
R-SMAD: Receptor-Regulated Smad
ROCK: Rho-like GTPase signaling pathway
ShcA: SRC homology 2–containing adaptor
Ser: Serine
SGS: Shprintzen-Goldberg syndrome
SMURF: Smad-specific E3 ubiquitin protein ligase
SOS: guanine nucleotide exchange factor for Ras
SRC: non-receptor tyrosine kinase
STK: Serine/Threonine kinase domain
TAAD: Thoracic Aortic Aneurysms and Dissections
TFGβ: Transforming Growth Factor-β
TGFBR1: TFGβ receptor 1
TGFBR2: TFGβ receptor 2
TGFBR3: TFGβ receptor 3
Thr: Threonine
Tyr: Tyrosine
vEDS: vascular Ehlers-Danlos Syndrome
WT: Wild-type
